# Frequent Attenders in a Psychiatric Emergency Department: A Descriptive Analysis

**DOI:** 10.1192/j.eurpsy.2025.1453

**Published:** 2025-08-26

**Authors:** R. Fernández-Fernández, L. Fontecha, C. Suárez, C. del Álamo

**Affiliations:** 1 Hospital Universitario Infanta Cristina, Parla, Spain

## Abstract

**Introduction:**

Frequent attenders (FAs) are defined as patients who repeatedly visit emergency services, commonly characterized as those making ≥4 visits to the emergency department within a year (Pek et al. Ann Acad Med Singap 2022; 51:483-492). Their identification is considered a potentially preventable misuse of resources (Pines et al. Acad Emerg Med 2011; 18).

**Objectives:**

To identify and describe the frequent attenders of the Psychiatric Emergency Department at Hospital Universitario Infanta Cristina.

**Methods:**

A descriptive analysis was carried out on all patients who made ≥4 visits to the emergency department at the hospital during 2023.

**Results:**

We identified 28 frequent attenders (FAs) in our emergency department, 16 women and 11 men, who made a total of 162 visits to the emergency service. Of these, 23 patients were aged between 18 and 65 years, with a mean age of 32.87 years. The most common diagnosis was Personality Disorders, observed in 17 patients, followed by Depression in 16 patients. However, the patients with the highest number of visits on average were those diagnosed with Psychosis, recording an average of 8.30 visits per year, followed by those diagnosed with Personality Disorders, with an average of 7.76 visits per year. No patients with Bipolar Disorder were identified among the described FAs.

When analyzing temporal trends, the months with the highest number of visits from FAs were July, August, and September, with 22, 18, and 19 visits respectively. This pattern is mirrored among patients diagnosed with Psychosis, who made 30 visits during these months. In contrast, patients with Personality Disorders made 28 visits in these months, compared to 24 visits in April and 30 visits in January and February.

An additional noteworthy finding is that, out of the 162 visits made by FAs, only 20 resulted in hospital admission—14 women and 6 men. More than half of these admissions involved patients with neurotic spectrum disorders, specifically 7 cases of Personality Disorders and 6 cases of Depression

Finally, it is worth noting that of the 162 visits made by FAs, 47 were due to suicidal ideation or attempts, with 29 of these cases involving women.

**Conclusions:**

The analysis of these patients suggests that individuals with severe mental disorders may be more affected during summer months, often requiring urgent evaluations, while those with neurotic spectrum disorders seem to be more influenced by seasonality, with a higher likelihood of requiring hospitalization. Additionally, there is a significant gender bias, with women tending to visit the emergency department more frequently, presenting with more severe conditions, and having a higher rate of hospital admissions. Accurate characterization of these patients can facilitate the prevention of potential decompensations by enhancing monitoring strategies based on the data presented.

**Disclosure of Interest:**

None Declared

